# *In Silico* Analysis on the Interaction of Haloacid Dehalogenase from *Bacillus cereus* IndB1 with 2-Chloroalkanoic Acid Substrates

**DOI:** 10.1155/2022/1579194

**Published:** 2022-10-08

**Authors:** Enny Ratnaningsih, Saepulloh Saepulloh

**Affiliations:** ^1^Biochemistry Research Division, Faculty of Mathematics and Natural Sciences, Bandung Institute of Technology, Bandung 40132, Indonesia; ^2^Center for Pulp and Paper, Ministry of Industry, Bandung 40258, Indonesia

## Abstract

Recently, haloacid dehalogenases have gained a lot of interest because of their potential applications in bioremediation and synthesis of chemical products. The haloacid dehalogenase gene from *Bacillus cereus* IndB1 (*bcfd1*) has been isolated, expressed, and Bcfd1 enzyme activity towards monochloroacetic acid has been successfully studied. However, the structure, enantioselectivity, substrate range, and essential residues of Bcfd1 have not been elucidated. This research performed computational studies to predict the Bcfd1 protein structure and analyse the interaction of Bcfd1 towards several haloacid substrates to comprehend their enantioselectivity and substrates' range. Structure prediction revealed that Bcfd1 protein consist of two domains. The main domain consists of seven *β*-sheets connected by six *α*-helices and four 3_10_-helices forming a Rossmannoid fold. On the other hand, the cap domain consists of five *β*-sheets connected by five *α*-helices. The docking simulation showed that 2-chloroalkanoic acids bind to the active site of Bcfd1 with docking energy decreases as the length of their alkyl chain increases. The docking simulation also indicated that the docking energy differences of two enantiomers of 2-chloroalkanoic acids substrates were not significant. Further analysis revealed the role of Met1, Asp2, Cys33, and Lys204 residues in orienting the carboxylic group of 2-chloroalkanoic acids in the active site of this enzyme through hydrogen bonds. This research proved that computational studies could be used to figure out the effect of substrates enantiomer and length of carbon skeleton to Bcfd1 affinity toward 2-chloroalkanoic acids.

## 1. Introduction

Haloacids compounds are known as toxic and harmful to many organisms, including animals and humans. These compounds, such as 2-chloropropionic acid and 2,2-dichloropropionic acid, were widely used as intermediate in agricultural and chemical industries [1, 2]. Haloacid dehalogenase catalyses the cleavage of the carbon-halogen bond in haloacid substrates to produce hydroxy acid and a halide [3], which means it is able to convert toxic haloacid to nontoxic compound. Some haloacid dehalogenases were known to be enantioselective and hence possess the potential to enrich the synthesis of certain hydroxy acid enantiomers or in the purification of a haloacid enantiomer from its racemic mixture [4]. Therefore, haloacid dehalogenases have gained great interests among researchers to be applied in bioremediation as well as in the synthesis of various chemical products [5–7].

Many bacteria were reported to produce haloacid dehalogenases, but only a few have been thoroughly studied to elucidate their structures, characteristics, and reaction mechanisms [8]. These enzymes can be divided into two groups according to conserved catalytic residues and reaction mechanism [9, 10]. Group I haloacid dehalogenase catalyses dehalogenation without formation of ester intermediate and consist of the nonenantioselective DL-haloacid dehalogenases [3, 11] and D-haloacid dehalogenases [12, 13]. Meanwhile, group II haloacid dehalogenase catalyses dehalogenation through the formation of ester intermediates and only has activity towards L-haloacid [14, 15].

Studies of haloacid dehalogenases showed that they have conserved essential residues in its catalytic site and determined substrate binding [7, 10] and enantioselectivity [4, 16]. Furthermore, the size of active site on haloacid dehalogenase affects the range of its substrates [17]. Hence, the enantioselectivity and substrate range of each haloacid dehalogenase could determine its potential application and a study on newly found haloacid dehalogenase is very important.

Haloacid dehalogenase from *Bacillus cereus* IndB1 (Bcfd1) was confirmed to have activity towards monochloroacetic acid [18] but its structure, enantioselectivity, substrate range, and essential residues have not been studied. Computational modelling and molecular docking have been used by other researchers as an alternative method to study the characteristics and interactions between haloacid dehalogenases and their substrates, such as DehD [19], DehE [3], and DehL [8]. This research reports in silico studies on Bcfd1 to predict its tertiary structure, important residues in its active site, and its interaction towards several 2-chloroalkanoic acids with various length of carbon skeleton.

## 2. Materials and Methods

### 2.1. Prediction of Tertiary Structure Bcfd1

The sequence of the haloacid dehalogenase gene from *B. cereus* IndB1 *(bcfd1)* [18] (GenBank accession number KU498039) was converted to amino acid sequence using Translate ExPASy (https://www.expasy.org/resources/translate). The Bcfd1 sequence was then aligned to UniProtKB_pdb database using BLASTP (https://www.uniprot.org/blast/) to search for templates for Bcfd1 structure prediction.

The prediction of the Bcfd1 tertiary structure was carried out using the ab initio and fold recognition or fragment assembly methods, applying I-Tesser [20] to give Bcfd1_01, Robetta [21] to result Bcfd1_02, TrRosetta [22] to produce Bcfd1_03, Robetta ab initio [23] to give Bcfd1_04, C-Quark [24] to produce Bcfd1_05, and RaptorX [25] to give Bcfd1_06. Visual molecular dynamics (VMD) were used to visualize and align all obtained Bcfd1 structural models to determine their similarities and differences. All six Bcfd1 models were evaluated in terms of 3D profile and geometrical aspects using ERRAT [26], Verify3D [27], PROVE [28], and QMEAN [29] as well as its stereochemistry using PROCHECK [30] and WHATCHECK [31].

### 2.2. Comparison of the Bcfd1 Tertiary Structure with Other Haloacid Dehalogenases

The best model of Bcfd1 tertiary structure was compared to all haloacid dehalogenase structures available in PDB using the DALI server [32]. VMD and MultiSeq were used for structural alignment to analyse active site residues and structure similarities.

### 2.3. Interaction of Bcfd1 with 2-Chloroalkanoic Acid Substrates

Molecular docking was carried out using nine 2-chloroalkanoic acids (haloacids) ligands, namely, monochloroacetic acid (MCA), L-2-chloropropionic acid (L2CP), D-2-chloropropionic acid (D2CP), L-2-chlorobutanoic acid (L2CB), D-2-chlorobutanoic acid (D2CB), L-2-chloropentanoic acid (L2CPn), D-2-chloropentanoic acid (D2CPn), L-2-chlorohexanoic acid (L2CH), and D-2-chlorohexanoic acid (D2CH). The structure of the nine ligands were prepared and minimized using the MarvinSketch program with the MMFF94 force field. The charges and polar hydrogen atoms were added to these nine ligands as well as to Bcfd1 using the AutoDock tool 1.5.7. Molecular docking was then performed using AutoDock Vina [33] employing the Lamarckian genetic algorithm (LGA) for searching the best pose of haloacids inside the active site of Bcfd1. The dimension of the grid box in the active site of Bcfd1 was determined to be 20 × 20 × 20 Å. The docking process was carried out by setting the exhaustiveness value to 24, the number of modes was 50 for each [34], and the simulation was repeated ten times for each ligand. The best docking pose for each ligand was determined according to the docking score (affinity energy) and its orientation in the active site. The interaction between each ligand and Bcfd1 residues were analysed using LigPlot+ [35].

## 3. Results and Discussion

### 3.1. Prediction and Evaluation of the Bcfd1 Tertiary Structure

Sequence alignment of Bcfd1 with proteins in UniProtKB_pdb database revealed that Bcfd1 possessed high sequence similarity to proteins belonging to haloacid dehalogenase (HAD) superfamily hydrolase; in which, according to InterPro protein families and domains [36], only L-haloacid dehalogenases that belong to this superfamily. This fact suggested that Bcfd1 might belong to HAD superfamily along with L-haloacid dehalogenase. The highest sequence similarity was obtained towards phosphatase from *Bacillus subtilis* 168 (YwpJ), though it only has 32.5% identity with *e*-value of 2.60*E *−* *46 indicating that the alignment is highly unique with no error. Hence, the tertiary structure of Bcfd1 was predicted using ab initio and fragment assembly methods.

**Figure 1:**
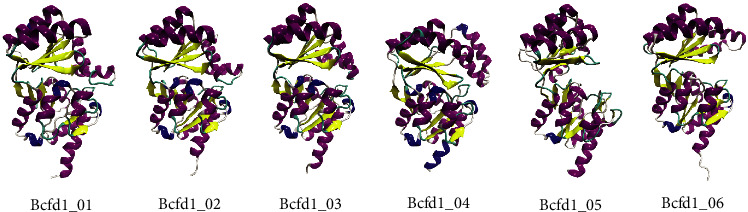
Predicted tertiary structure models of Bcfd1. Bcfd1_01, Bcfd1_02, Bcfd1_03, Bcfd1_04, Bcfd1_05, and Bcfd1_06 were obtained using I-Tasser, Robetta, TrRosetta, Robetta ab initio, C-Quark, and RaptorX, respectively. All models possessed similar main domain with slightly different on its cap domain.

Prediction of the Bcfd1 tertiary structure using six web services produced six models that are best models from these web services. These six predicted models are presented in Figure 1. All Bcfd1 models revealed to have main and cap domain that consists of several *α*-helices and *β*-sheets forming the Rossmannoid fold. Structural alignment analysis on these Bcfd1 models showed significant differences on the cap structural domain. The range of the root mean square deviation (RMSD) of all Bcfd1 models was 1.399–3.326 Å, indicating that they were slightly different from each other. Therefore, the evaluation of all Bcfd1 models needs to be further conducted to choose the best model to be used for further analysis on its interaction with haloacid ligands.

Evaluation on 3D structural models of Bcfd1 using ERRAT, Verify3D, PROVE, and QMEAN are represented as its scores in Table 1. ERRAT evaluates the quality of all our Bcfd1 structural models based on statistics of nonbonded atom-atom interactions (C-C, C-N, N-N, N-O, and O-O) which then compared to a database of reliable high-resolution structures [26]. The ERRAT score above 50% indicates good structural quality [37]. Hence, all our Bcfd1 models could be considered as good models.

Verify3D is used to check the compatibility of the 3D structural model of Bcfd1 based on the location of its residues and compared to protein structures found in PDB [27, 38]. Verify3D scores above 80% categorizes the protein as good quality structure [37], which was true for our Bcfd1 structural models.

PROVE evaluates the quality of Bcfd1 structural model based on volume deviations of atoms in the protein structure from the value of standard atomic volume (volume *Z*-score) [28]. PROVE calculates the percentage of atoms in the buried protein region that has three times greater volume compared to the standard deviation of this type of atom (outlier). The data in Table 1 show that Bcfd1_03 has the smallest percentage of outlier atoms, indicating this structure is a better model compared to other. Bcfd1_03 also has the highest QMEAN score. Therefore, the Bcfd1_03 model was considered as the best model in terms of 3D profile and geometrical aspects.

The stereochemistry evaluation of all Bcfd1 structural models using PROCHECK and WHATCHECK are presented in Tables 2 and 3, respectively. The PROCHECK assesses the stereochemical quality of the Bcfd1 structural model based on its amino acids geometric analysis compared to the high-resolution protein structure and displays it in the form of a Ramachandran plot [30]. The result showed that Bcfd1_06 has the highest number of residues in the core region of the Ramachandran plot, followed by Bcfd1_03, which indicates that these two structures have the highest number of amino acids in favoured conformation; hence, they are more stable than others. However, the Bcfd1_03 is considered as a better model since it has no residues in the disallowed region.

On the other hand, WHATCHECK is used to perform more extensive calculations on stereochemical parameters [31]. WHATCHECK results are grouped into structure *Z*-score and RMS *Z*-score. The positive value of structure *Z*-score indicates a better structure, while the value of the RMS *Z*-score is better if it is close to one. The summary of WHATCHECK evaluation showed that, in terms of structure *Z*-score, the Bcfd1_03 model has the best scores compared to other models. Furthermore, in term of the RMS *Z*-score, only Bcfd1_02, Bcfd1_03, and Bcfd1_04 have scores close to 1. These analyses indicated that Bcfd1_03 model was the best stereochemistry model and therefore used for further analysis.

### 3.2. Tertiary Structure Comparison of Bcfd1 with Other Haloacid Dehalogenases

**Figure 2:**
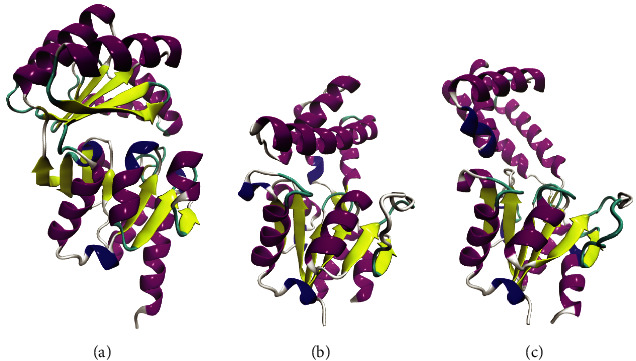
The tertiary structure comparison of (a) Bcfd1 with (b) L-DEX YL from *Pseudomonas* sp. YL and (c) ps-2-HAD from *P. syringae*. Bcfd1 has a main domain similar to the main domain of ps-2-HAD and L-DEX YL but the cap domain is significantly different.

This analysis was performed using DALI server [39] to find haloacid dehalogenase that has a similar structure to Bcfd1_03 (Figure 1), particularly for the active site and enantioselectivity prediction of Bcfd1. General analysis towards all PDB protein structures suggested that Bcfd1_03 (from now on called Bcfd1) is similar to L-haloacid dehalogenases of some bacteria as presented in Table 4. Moreover, Bcfd1 also showed similarity to DL-haloacid dehalogenase from *P. syringae* pv. Tomato DC3000 (ps-2-HAD) [17]. Therefore, further detail analysis was performed by aligning Bcfd1 structure with L-haloacid dehalogenase (L-DEX YL) [40] and ps-2-HAD to figure out structural similarities and differences. The main domain of both Bcfd1 and L-DEX YL consisted of several *α*-helices and *β*-sheets forming the Rossmannoid fold; however, Bcfd1 has more *α*-helices and *β*-sheets than L-DEX YL. Furthermore, the Bcfd1 cap domain consists of five *β*-sheets connected by five *α*-helices and located between the fourth and the fifth *β*-sheet of the main domain, whereas the cap domain of L-DEX YL consisted only four helices that located between the first and the second *β*-sheets of the main domains. The comparison of Bcfd1 to ps-2-HAD was also similar, as L-DEX YL possessed high similarity to ps-2-HAD. The tertiary structure of Bcfd1, L-DEX YL, and ps-2-HAD are shown in Figure 2.

**Figure 3:**
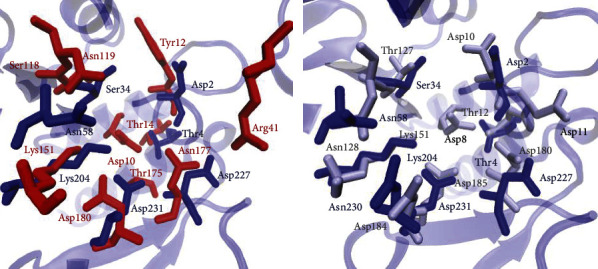
Active site alignment of Bcfd1 (blue) with L-DEX YL (red) from *Pseudomonas* sp. YL and ps-2-HAD (gray) from *P. syringae*. The active site of Bcfd1 is more similar to the active site of ps-2-HAD with more aspartate residues than L-DEX YL which is predicted to be involved in the catalytic reaction mechanism.

To identify whether Bcfd1 has conserved residues essential for activity as in L-DEX YL and ps-2-HAD, further detail tertiary structural alignment was conducted (Figure 3). Alignment results revealed that Bcfd1 has almost conserved residues compared to L-DEX YL, which were identified to be essential in substrates binding (Thr4, Ser34, Asn58, Lys204, and Asp227) and performing hydrolysis (Asp180) (digit indicates the position of the residue in the protein sequence). These residues were almost superimposed with Asp2, Thr4, Ser34, Asn58, Lys204, Asp227, and Asp231 in Bcfd1. However, compared to L-DEX YL, Bcfd1 does not have conserved Asp10 and Arg41 that acts as nucleophilic residue and essential for stabilizing the halide atom, respectively.

Compared to ps-2-HAD, Bcfd1 showed the most essential residues except Asp8 which was predicted to be involved in catalytic reactions. Both Bcfd1 and ps-2-HAD do not have conserved arginine residues as in the most L-haloacid dehalogenases. Nevertheless, Bcfd1 and ps-2-HAD have more aspartate residues in the active site compared to L-haloacid dehalogenase [17]. Therefore, the active site residues of Bcfd1 are more similar to ps-2-HAD than L-haloacid dehalogenases. Similarities and differences of these residues in active site of each enzyme are presented in Figure 3.

### 3.3. Molecular Docking of 2-Chloroalkanoic Acids in Bcfd1

Molecular docking of 2-chloroalkanoic acids in the active site of Bcfd1 was performed to identify the effect of substrates enantiomer and length of carbon skeleton to Bcfd1 affinity. The molecular docking average results from ten experiments showed that docking energy decreases as the length of the alkyl chain in ligand increases (Table 5). Our docking results were in good agreement with those reported by Schmidberger et al. [10], in which ligands with longer alkyl chains tend to have lower calculated binding energies (affinities). However, the visualization of each haloacid pose with the lowest energy indicated that only MCA, L2CP, and D2CP were bound to the active site of Bcfd1. Ligands with more than three carbon atoms skeleton were bound to another binding pocket of Bcfd1, though could still be bound in the active site of Bcfd1 with higher energy (lower affinity). Therefore, Bcfd1 was estimated to have lower activity towards 2-chloroalkanoic acids with the skeleton of more than three carbon atoms.

Data in Table 5 indicated that docking energy (affinity) of L-haloacids were not significantly different from D-haloacids. These results were in line with the previous experiment reported by Hamid et al. [3] who stated that binding free energy of L2CP was not different from D2CP in DL-haloacid dehalogenase of *Rhizobium* sp. RC1 (DehE). The results of these analyses suggested that Bcfd1 was active towards both L2CP and D2CP, which also observed in wet experiment of ps-2-HAD for *P. syringae* pv. Tomato DC3000 [17]. However, Schmidberger et al. [10] reported that both L- and D-ligands could be bound in the active site of L-haloacid dehalogenase from *B. cepacea* (DehIVa) without significant difference of docking energy (affinity) although wet experiment only observed its activity towards L-ligands. Therefore, further experiments still need to be performed to confirm Bcfd1 enantioselectivity.

**Figure 4:**
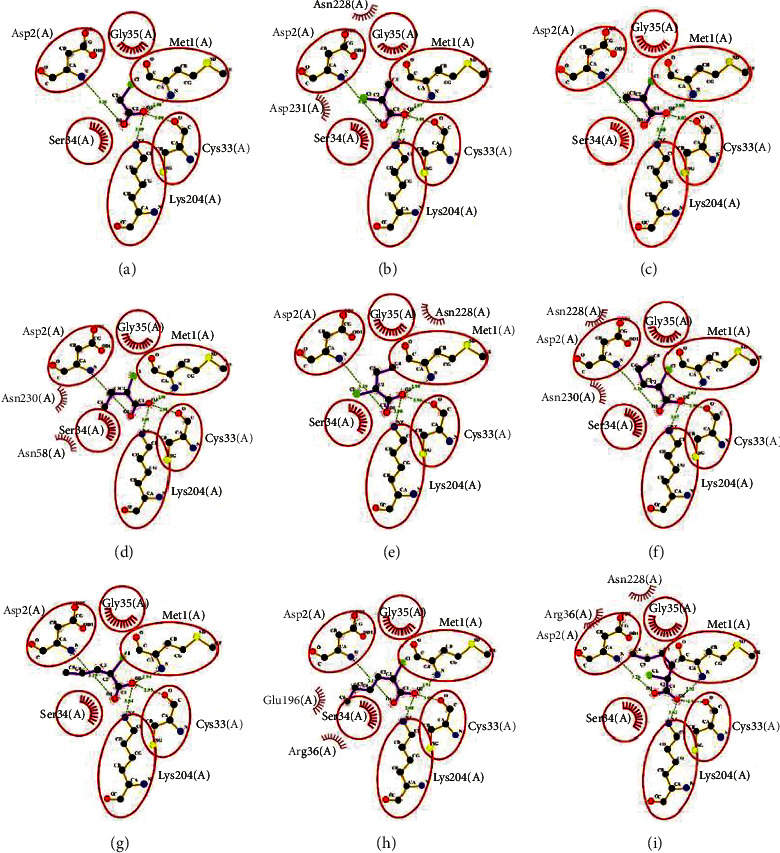
Schematic interaction of residues in the active site of Bcfd1 with chloroalkanoic acids. (a) MCA. (b) L2CP. (c) D2CP. (d) L2CB. (e) D2CB. (f) L2CPn. (g) D2CPn. (h) L2CH. (i) D2CH. Four residues (Met1, Asp2, Cys33, and Lys204) form hydrogen bonds with ligands orienting its carboxylate groups to the same direction that indicates the important role of these four residues of the Bcfd1 active site.

Analyses on the interaction between residues of Bcfd1 with nine different 2-chloroalkanoic acids using LigPlot+ revealed that Met1, Asp2, Cys33, and Lys204 form hydrogen bonds with ligands (Figure 3). These hydrogen bonds oriented carboxylic groups of all ligands to the active site of Bcfd1. Furthermore, LigPlot+ also figures out that hydrophobic interaction between residues and ligands were obtained to be performed by Gly35, Ser34, Asn58, Asn228, Asn230, and Asp231. The number of residues that form hydrophobic interactions with haloacids tends to increase as the number of carbon atoms in the haloacids increases (Figure 4). This analysis indicated the important role of some residues in binding and orienting ligands especially Met1, Asp2, Cys33, and Lys204 [40].

## 4. Conclusions

Results from this study clearly indicated that haloacid dehalogenase (Bcfd1) produced by *Bacillus cereus* IndB1 belongs to HAD superfamily along with L-haloacid dehalogenase. Structurally, the cap domain of Bcfd1 was similar to L-DEX YL from *Pseudomonas* sp. YL and to ps-2-HAD from *P. syringae* with conserved substrate binding residues, but its cap domain was significantly different. Though ligands up to six carbon skeletons could bind to the Bcfd1 active site, its docking energy revealed to decrease as the length of the alkyl chain increases. The carboxylic groups of all ligands were found to be oriented to the active site by forming hydrogen bonds. Further experiments are still imperatively needed to analyse the interaction and stability of Bcfd1-ligand complex using molecular dynamics and wet experiments are also needed to study Bcfd1 activity toward all of the substrates as well as to confirm its enantioselectivity.

## Figures and Tables

**Table 1:** The 3D profile and geometrical aspects evaluation of the Bcfd1 structure models.

No	Model	ERRAT	Verify3D (%)	PROVE (%)	QMEAN
1	Bcfd1_01	96.00	82.33	5.50	−3.18
2	Bcfd1_02		90.11	3.90	1.72
**3**	**Bcfd1_03**	**99.27**	**93.64**	**3.20**	**1.86**
4	Bcfd1_04		85.87	4.20	0.86
5	Bcfd1_05	90.91	100.00	5.90	−6.25
6	Bcfd1_06	94.49	85.87	11.60	1.14

**Table 2:** Bcfd1 models evaluation using PROCHECK.

No	Model	*Ramachandra plot*				Chi1-chi2 plots	Bad contacts	*Planar groups*		
		Core (%)	Allow (%)	General	Disallowed			Within limits (%)	High lighted	Off graph
1	Bcfd1_01	81.00	15.90	1.20%	1.90%	14	5	82.50	17.50%	9
2	Bcfd1_02	90.70	9.30	0	0	2	2	99.00	1.00%	0
**3**	**Bcfd1_03**	**93.00**	**7.00**	**0**	**0**	**2**	**1**	**100**	**0**	**0**
4	Bcfd1_04	90.70	8.90	0.40%	0	1	0	100	0	0
5	Bcfd1_05	76.40	15.10	4.30%	4.30%	15	1	82	18.40%	5
**6**	**Bcfd1_06**	**93.80**	**5.80**	**0**	**0.40%**	**2**	**8**	**100**	**0**	**0**

**Table 3:** Summary of Bcfd1 models evaluation using WHATCHECK.

No	Model	*Structure Z-scores (positive is better than average)*			*RMS Z-scores (should be close to 1.0)*			
		1st generation packing quality	Ramachandran plot appearance	chi1/chi2 rotamer normality	Bond lengths	Bond angles	Omega angle restraints	Side chain planarity
1	Bcfd1_01	−0.631	−3.654	−3.605	0.62	1.383	2.383	1.623
2	Bcfd1_02	−0.208	-0.305	2.672	**0.737**	**0.907**	**0.777**	**0.744**
3	Bcfd1_03	**1.117**	**1.110**	**4.636**	**0.708**	**0.825**	**0.524**	**0.518**
4	Bcfd1_04	−0.062	−0.892	3.301	**0.767**	**0.962**	**0.603**	**0.687**
5	Bcfd1_05	−1.091	−4.414	−3.018	0.652	1.514	2.817	2.155
6	Bcfd1_06	**1.128**	**0.832**	**2.566**	3.888	2.247	1.026	0.028

**Table 4:** The structure alignment results of Bcfd1 with the haloacid dehalogenases using the DALI server.

No.	PDB	Organism	Protein	*Z*-score	Identity (%)	Residues aligned	RMSD (Å)	Reference
		*Bacillus cereus*	Bcfd1					
1	3vay_A	*Pseudomonas syringae*	Ps-2-HAD	10,7	13	120	2, 6	[17]
2	3um9_A	*Polaromonas* sp. JS666	—	10,5	12	125	2, 6	—
3	1zrm_A	*Pseudomonas* sp. YL	L-DEX YL	10,4	12	119	2, 4	[40]
4	1jud_A	*Pseudomonas* sp. YL	L-DEX YL	10,3	10	124	2, 5	[41]
5	2w43_A	*Sulfolobus tokodaii*	—	10,1	[42]
6	1zrn_A	*Pseudomonas* sp. YL	L-DEX YL	10,2	11	124	2, 5	[40]
7	4cf3_A	*Rhodobacteraceae*	DehRhb	9,9	13	127	3, 0	—
8	2yn4_A	*Rhodobacteraceae*	DehRhb	9,7	13	130	3, 0	[7]
9	4cnq_A	*Rhodobacteraceae*	DehRhb	9,7	14	130	3, 0	—
10	1qq5_A	*Xanthobacter autotrophicus*	DhlB	9,6	15	128	2, 8	[43]
11	1qq6_A	*Xanthobacter autotrophicus*	DhlB	9,6	13	129	2, 8	[43]
12	4ce6_A	*Rhodobacteraceae*	DehRhb	9,6	15	129	3, 0	—
13	1qq7_A	*Xanthobacter autotrophicus*	DhlB	9,6	14	129	2, 8	[43]
15	2no5_A	*Burkholderia cepacia*	DehIVa	9,3	15	127	2, 8	[10]
16	1aq6_A	*Xanthobacter autotrophicus*	DhlB	9,2	14	127	2, 8	[44]
17	2no4_A	*Burkholderia cepacia*	DehIVa	9,2	14	125	2, 7	[10]
18	2w11_A	*Sulfolobus tokodaii*	—	9,1	14	126	2, 9	[42]
19	1qh9_A	*Pseudomonas* sp. YL	L-DEX YL	8,9	11	124	2, 5	—
20	3umg_A	*Rhodococcus jostii* RHA1	—	8,8	11	132	3, 3	—
21	2ymp_A	*Rhodobacteraceae*	DehRhb	8,8	14	128	3, 1	[7]
22	2ymm_A	*Rhodobacteraceae*	DehRhb	8,7	13	126	2, 9	[7]
23	3umb_A	*Ralstonia solanacearum*	—	8,6	14	122	2, 5	—
24	4cf5_A	*Rhodobacteraceae*	DehRhb	8,6	11	124	2, 9	—
25	4cf4_A	*Rhodobacteraceae*	DehRhb	8,5	13	129	2, 9	—
26	2yml_A	*Rhodobacteraceae*	DehRhb	8,4	13	129	3, 0	[7]
27	3umc_A	*Pseudomonas aeruginosa*	—	8,4	9	127	3, 0	—
28	2ymq_A	*Rhodobacteraceae*	DehRhb	8.3	13	128	3.0	[7]

**Table 5:** Docking energy average of 2-chloroalkanoic acids in the active site of Bcfd1.

No	Ligand	Code	Affinity (kkal/mol)	RMSD (Å)	Pose
1	Monochloroacetic acid	MCA	−3.6	0–0.03	Mode 1
2	L-2-chloropropionic acid	L2CP	−4.1	0	Mode 1
3	D-2-chloropropionic acid	D2CP	−4.1	0	Mode 1
4	L-2-chlorobutanoic acid	L2CB	−4.2	0–0.04	Mode 4
5	D-2-chlorobutanoic acid	D2CB	−4.3	0.03–0.04	Mode 5
6	L-2-chloropentanoic acid	L2CPn	−4.4	0.02–0.06	Mode 3
7	D-2-chloropentanoic acid	D2CPn	−4.5	0,01–0,04	Mode 4
8	L-2-chlorohexanoic acid	L2CH	−4.6	0.05–0.09	Mode 4
9	D-2-chlorohexanoic acid	D2CH	−4.7	0.02–0.14	Mode 4

## Data Availability

All of the data used in this paper could be further access in Institut Teknologi Bandung library as magister thesis under the name of Saepulloh.
